# Determinants of oral pathogen prevalence in North-East Nigeria: a cross-sectional study

**DOI:** 10.1186/s12903-026-08774-w

**Published:** 2026-06-01

**Authors:** Auwal Magaji, Fatima Magaji Ibrahim

**Affiliations:** 1 Department of Microbiology, Federal University of Health Sciences, Azare, Bauchi State Nigeria; 2 Department of Health Education, Aminu Saleh College of Education, Azare, Bauchi State Nigeria

**Keywords:** Caries, Dental, Flossing, Gum bleeding, Oral hygiene, Oral pathogens

## Abstract

**Background:**

Oral diseases remain a major public health problem globally, with limited data available from North-East Nigeria. This study assessed the prevalence of oral pathogens and their risk factors among patients in North-East Nigeria.

**Methods:**

A hospital-based cross-sectional study was conducted between March and August 2025, among 300 patients with oral health complaints in selected health facilities. Data were collected using structured questionnaires. Oral swabs were collected, processed and assessed for pathogens using standard microbiological techniques. Statistical analyses were performed using chi-square tests and logistic regression to identify predictors of oral pathogens’ colonization, with *p* < 0.05 considered significant.

**Results:**

Overall, 37.67% of participants were positive for at least one oral pathogen. The most prevalent bacteria detected were *Staphylococcus aureus* (16.33%) and *Streptococcus mutans* (12.33%). *Candida albicans* (4.67%) was only the fungal pathogen detected. Females (22.00%) and urban residents (23.33%) exhibited significantly higher prevalence than males (15.67%) and rural residents (14.34%). Brushing frequency (χ² = 11.74, *p* < 0.01) and flossing (χ² = 8.31, *p* = 0.02) were significantly associated with lower prevalence of oral pathogens. Female gender (OR = 5.62; 95% CI: 3.35–9.41; *p* < 0.01), urban residence (OR = 2.10; 95% CI: 1.26–3.50; *p* = 0.01), chronic illness (OR = 5.19; 95% CI: 1.01–26.6; *p* = 0.03), and poor flossing practice (OR = 0.52; 95% CI: 0.30–0.90; *p* = 0.01) are associated with high odds of oral pathogens detection.

**Conclusion:**

The study revealed a high prevalence of oral pathogens among patients with oral problems in North-East Nigeria, with *S. aureus* and *S. mutans* as the predominant isolates. Poor oral hygiene practices, female gender, and area of residence were associated with high risk of the pathogens colonization. Strengthening oral health education, promoting regular tooth brushing and flossing, and improving access to preventive dental services could help in reducing the burden of oral pathogens in the region.

**Supplementary Information:**

The online version contains supplementary material available at 10.1186/s12903-026-08774-w.

## Introduction

The human oral cavity harbors one of the most diverse microbial ecosystems in the body, comprising over 700 species of bacteria, fungi, and viruses [1]. Under normal conditions, these microorganisms exist in a dynamic equilibrium with the host and contribute to oral and systemic health [2]. However, disruption of this balance due to poor oral hygiene, dietary habits, or host-related factors may promote microbial dysbiosis, allowing pathogenic organisms to proliferate [2]. Such imbalances have been associated with common oral diseases, including dental caries, gingivitis, and periodontitis, and may also contribute to systemic conditions such as diabetes, cardiovascular diseases, and respiratory infections [1, 3].

Beyond the biological mechanisms, oral diseases represent a major global public health challenge. It is estimated that approximately 3.5 billion people are affected worldwide, making oral diseases among the most prevalent non-communicable conditions [4]. In Nigeria, the burden is similarly substantial. The prevalence of dental caries among adults ranges from 4% to 40%, with a high proportion of untreated cases [5]. Among children, early childhood caries affects about 17% [6]. These findings highlight the widespread and persistent nature of oral health problems across different population groups.

Dietary habits and lifestyle factors contribute significantly to the increasing burden of dental problems [7]. Poor oral hygiene practices remain a key driver of this burden. Studies in rural communities in Edo and Delta States have shown that over one-third of individuals exhibit poor oral hygiene, with many brushing only once daily and a large proportion never having visited a dentist [8]. Similarly, national data indicate low levels of optimal oral hygiene practices among adolescents, characterized by irregular tooth brushing, low flossing rates, and high consumption of sugary foods [9]. Among pregnant women, oral care practices are often inconsistent, with continued reliance on traditional cleaning methods such as chewing sticks [10].

Importantly, the distribution and prevalence of specific oral pathogens play a central role in the development and progression of oral diseases. For example, *Streptococcus mutans* is strongly associated with dental caries, particularly in individuals with high sugar intake and poor oral hygiene, while *Candida albicans* is more common among immunocompromised individuals and those with inadequate oral care [1]. Understanding the prevalence and distribution of these pathogens is therefore critical for identifying at-risk populations, guiding targeted preventive strategies, and improving oral health outcomes.

In addition to biological and behavioral factors, sociocultural and structural determinants significantly influence oral health in Nigeria. Cultural beliefs and practices related to oral hygiene may affect the adoption of preventive measures [11]. Furthermore, limited access to dental care services, poverty, and low awareness contribute to the persistence of oral diseases, particularly in underserved regions such as North-East Nigeria.

Despite the growing recognition of oral health as a public health priority, there is limited data on the prevalence of oral pathogens and their associated factors in North-East Nigeria. Generating such evidence is essential for informing context-specific interventions and strengthening oral health policies in the region.

This study aims to determine the prevalence of oral pathogens, identify associated factors, and assess the relationship between oral hygiene practices and oral pathogen occurrence among patients in North-East Nigeria.

## Methodology

### Study area and design

**Fig. 1:**
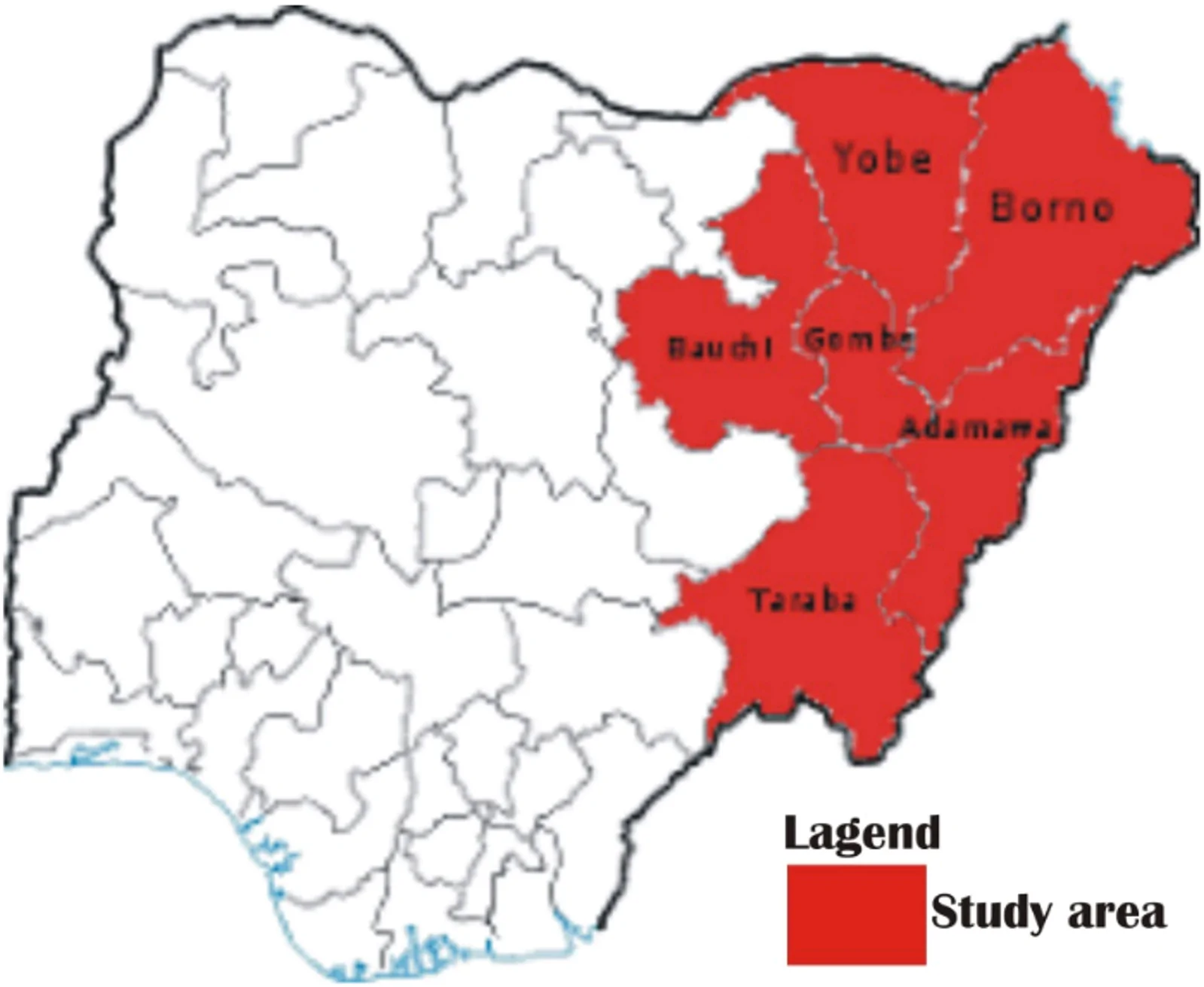
Map of Nigeria showing the study locations (Bauchi and Gombe States) in North-East Nigeria. The map was adapted from publicly available sources

This hospital-based cross-sectional study was conducted at Extreme Hospital Azare, Bauchi State, and Federal Medical Center Gombe State, Northeastern Nigeria between March and August, 2025. Data were collected from the study participants at single point in time. These states were chosen to provide a representative overview of oral health challenges in the region, which is characterized by limited dental health infrastructure, diverse sociocultural practices, and high burden of communicable diseases (Fig. 1). The map illustrating the study locations was adapted from publicly available sources.

### Study population

The study population comprised patients of all ages and genders who presented to the selected health facilities with clinical complaints related to oral health, including but not limited to dental pain, gum bleeding, oral ulcers, or suspected oral infections.

Participants were recruited using a convenience sampling method, whereby all eligible patients presenting during the study period and consenting to participate were consecutively enrolled until the required sample size was achieved.

Inclusion criteria included individuals who provided informed consent (or assent/parental consent for minors) and agreed to provide oral samples for laboratory analysis.

Exclusion criteria included patients who were critically ill, those currently undergoing intensive antimicrobial therapy that could interfere with microbial recovery, and individuals unable or unwilling to provide samples.

### Sample size determination

The sample size was calculated using the standard single-proportion formula. A prevalence (p) of 0.23 (23%) was adopted based on previously reported prevalence of oral health conditions in Nigeria, to provide a conservative estimate for the study population [5]. A precision (d) of 0.05 and a confidence level of 95% (Z = 1.96) were used.

$$\mathrm{n}=\frac{{\mathrm{Z}}^{2}\:X\:P(1-\:P)}{{d}^{2}}$$

$$\mathrm{Therefore},\:\mathrm{n}=\frac{{\left(1.96\right)}^{2}\:X\:0.23(1-\:0.23)}{{0.05}^{2}}$$

The minimum calculated sample size was 272. To account for potential non-response, incomplete data, and to improve statistical power, a 10% adjustment was applied, resulting in a final sample size of approximately 300 participants.

### Ethical considerations

Ethical approval was obtained from the Research and Ethics Committees of the Federal Medical Centers Gombe with reference numbers NHREC/25/10/2013 dated 3rd March, 2025, and Extreme Hospital Azare Bauchi State (reference numbers; EXHA/02/25/033). Written informed consent was obtained from adult participants, and from the parents or guardians of children below 16 years of age, before enrolment. Assent was also obtained from children aged seven years and above. Confidentiality of participants’ information was ensured, and all procedures were conducted in accordance with the Declaration of Helsinki (2013).

### Data collection

Socio-demographic information and oral hygiene practices were collected using a structured questionnaire developed by the research team (Supplementary File 1), administered through face-to-face interviews. The questionnaire captured variables such as age, gender, residence, oral hygiene habits (frequency of brushing, use of toothpaste, flossing, and traditional methods), dietary habits, and dental care utilization.

The questionnaire was developed after an extensive review of relevant literature on oral pathogenic bacteria, associated risk factors, oral hygiene practices, and antibiotic use behaviors. To ensure content validity, the initial draft of the instrument was reviewed by a panel of experts comprising microbiologists, dental public health specialists, and epidemiologists from Federal University of Health Sciences, Azare, Bauchi State, Nigeria. The experts assessed the questionnaire for relevance, clarity, comprehensiveness, and appropriateness of each item in addressing the study objectives. Their feedback led to modifications, including rephrasing ambiguous questions and removal of redundant items.

The reliability of the questionnaire was assessed through a pilot study conducted among the pretest participants (*n* = 30) drawn from a population similar to the study setting but not included in the final analysis. Participants were asked to comment on the clarity, wording, and understanding of the questions. A Cronbach’s alpha value of ≥ 0.76 was considered acceptable for reliability. The final version of the questionnaire demonstrated satisfactory internal consistency and stability, confirming its suitability for use in the main study.

In this study, “Recent antibiotic use” was defined as the use of systemic antibiotics within the past four weeks prior to sample collection.

Also, “Mouth sores/ulcers” were defined as visible lesions in the oral cavity, including ulcers, erosions, or inflamed mucosal areas, as reported by participants or observed during sample collection.

### Sample collection

Oral swab samples were collected aseptically from each participant by trained personnel using sterile cotton-tipped swab sticks. To ensure consistency and adequate recovery of oral pathogens, samples were obtained from standardized intraoral sites, including the buccal mucosa, tongue dorsum, gingival margin, and any visible lesion (such as ulcers, plaques, or inflamed areas) where present. In participants with suspected dental caries or periodontal involvement, swabs were also gently collected from the tooth surface and gingival crevice.

Prior to sample collection, participants were instructed to avoid eating, drinking, or oral hygiene practices (e.g., brushing or mouthwash use) for at least 1–2 h to minimize transient contamination. Each swab was rotated firmly across the sampling site for approximately 5–10 s to ensure adequate specimen collection. Immediately after collection, swabs were placed into sterile tubes containing Amies transport medium to preserve the viability of both fastidious bacterial and fungal organisms during transit. All samples were properly labeled with unique identifiers corresponding to the participant’s questionnaire data.

The samples were transported to the microbiology laboratories of the respective study centers (Federal Medical Centre Gombe and Extreme Hospital Azare) within 2–4 h of collection, maintained under cool conditions (4–8 °C) using insulated cold boxes. Where immediate processing was not possible, samples were temporarily stored at 4 °C and processed within 24 h to prevent overgrowth or loss of viable organisms.

Strict aseptic techniques were maintained throughout the collection and transport process to avoid contamination and ensure reliability of microbiological findings.

### Microbiological identification and quality control

Bacterial isolates were identified using standard microbiological procedures, including colony morphology, Gram staining, and biochemical characterization as described by Cheesbrough [12]. Morphological assessment included colony size, shape, elevation, pigmentation, and hemolytic patterns on blood agar.

Gram staining was performed to differentiate organisms into Gram-positive and Gram-negative bacteria. Subsequent biochemical identification involved catalase, coagulase, oxidase, and other relevant tests depending on the suspected organism. For instance, *Staphylococcus aureus* was identified based on Gram-positive cocci in clusters, catalase positivity, and coagulase positivity, while *Streptococcus mutans* was identified based on Gram-positive cocci in chains, catalase negativity, and characteristic growth patterns.

Fungal isolates were identified based on macroscopic and microscopic morphology, including colony appearance on Sabouraud dextrose agar, lactophenol cotton blue staining, and germ tube testing for *Candida albicans*. Where necessary, subculturing on Chromagar Candida was performed for presumptive differentiation [13].

To ensure reliability of laboratory findings, quality control measures were implemented. Culture media were prepared according to manufacturer instructions and checked for sterility and performance prior to use. Standard laboratory protocols were strictly followed for staining and biochemical tests. Known reference strains (where available) and previously characterized isolates were used to verify test performance and ensure consistency of identification procedures.

Furthermore, selected samples were processed in duplicate to confirm consistency of culture results, and all procedures were carried out following standard microbiological guidelines.

It is important to note that several of the isolated microorganisms, such as *Staphylococcus aureus* and *Streptococcus mutans*, may also exist as part of the normal oral microbiota. In this study, organisms were classified as potential pathogens based on their known association with oral diseases in the literature and their recovery from participants presenting with oral health complaints.

### Data analysis

Data were entered into SPSS version 23.0 (IBM Corp., Armonk, NY, USA) for statistical analysis. Descriptive statistics were used to summarize demographic characteristics, oral hygiene practices, and prevalence of pathogens. Associations between risk factors and occurrence of oral pathogens were assessed using chi-square tests, and multivariable logistic regression was applied to identify independent predictors. A *p*-value < 0.05 was considered statistically significant.

Although multiple variables were included in the regression model, some potential confounders such as detailed socioeconomic status, dietary sugar intake, prior dental treatment history, sanitation conditions, and quality of oral hygiene techniques were not comprehensively assessed.

Data were checked for completeness prior to analysis. Cases with missing or incomplete data were excluded from specific analyses where necessary (complete case analysis), and no imputation was performed.

## Results

### Demographic characteristics and prevalence of oral pathogens

Table 1 presents prevalence stratified by demographics. Prevalence rates were significantly higher (χ² = 13.88, *p* < 0.01) among adolescents aged 11–19 years (17.33%) and adults aged 25–64 years (17.33%) compared with younger children (1.67%). Females exhibited a significantly higher prevalence (22.00%) than males (15.67%) (χ² = 16.78, *p* < 0.01). Similarly, individuals residing in urban areas (23.33%) had a significantly higher prevalence than rural dwellers (14.34%) (χ² = 7.81, *p* = 0.01).

**Table 1:** Prevalence of oral pathogens by demographic characteristics

Stratum	*N* tested	*n* Positive	Prevalence (%)	95% CI	χ²	*p* value
Age						
Below 10 years	22	5	1.67	0.71–3.80	13.88	0.00*
11–19 years	102	52	17.33	13.33–21.81		
20–24 years	19	4	1.33	0.03–2.61		
25 64 years	160	52	17.33	13.33–21.81		
Gender						
Female	104	66	22.00	17.50–26.77	16.78	0.00*
Male	199	47	15.67	11.87–20.02		
Residence						
Rural	86	43	14.33	10.71–18.57	7.81	0.01*
Urban	217	70	23.33	18.71–28.16		

“*” = Significant at *p* < 0.05

### Prevalence and distribution of oral pathogens among the study participants

Out of the 300 oral samples analyzed, 113 (37.67%; 95% CI: 30.60–41.40) were positive for at least one pathogen (Table 2). *Staphylococcus aureus* was the most frequently isolated organism (16.33%; 95% CI: 12.20–20.40), followed by *Streptococcus mutans* (12.33%; 95% CI: 8.70–16.00), *Candida albicans* (4.67%%; 95% CI: 2.50–7.20), and *Staphylococcus epidermidis* (4.33%; 95% CI: 2.30–6.80).

**Table 2:** Prevalence of oral pathogens isolated from study participants (*N* = 300)

Pathogens	*N* tested	*n* positive	Prevalence %	95% CI
*Staphylococcus aureus*	300	49	16.33	12.20–20.40
*Staphylococcus mutans*	300	37	12.33	8.70–16.00
*Candida albicans*	300	14	4.67	2.50–7.20
*Staphylococcus epidermidis*	300	13	4.33	2.30–6.80
Total		113	37.67	30.60–41.40

“χ² “ = Chi-square

“*” = Significant at *p* < 0.05

### Oral hygiene practices and occurrence of oral pathogens

As shown in Table 3, brushing frequency was significantly associated with microbial colonization of the oral cavity (χ² = 11.74, *p* < 0.01). Those who brushed more than twice daily had the lowest proportion of the microbes. Flossing practice also showed significant association (χ² = 8.31, *p* = 0.02), with daily flossers recording the lowest proportion of positive cases. In contrast, type of cleaning material, toothbrush replacement, and mouth rinsing after meals did not show statistically significant associations (*p* > 0.05).

**Table 3:** Association between oral hygiene practices and occurrence of oral pathogens

Variables	Negative	Positive	Total	χ²	Df	*p* value
Cleaning material used						
Toothbrush and toothpaste	182	108	290	0.71	1	0.40
Chewing stick	5	5	10			
Charcoal/ash	0	0	0			
Brushing frequency						
Once daily	108	53	158	11.74	2	0.00*
Twice daily	82	54	136			
More than twice daily	0	6	6			
Toothbrush replacement						
Monthly	40	35	75	7.45	3	0.06
Every 3 months	76	32	108			
Every 6 months	21	19	40			
Only when worn out	50	27	77			
Flossing practice						
No	93	40	133	8.31	2	0.02*
Occasionally	53	39	92			
Yes, daily	41	34	75			
Mouth rinsing after meals						
Always	83	38	121	4.78	2	0.09
Never	2	0	2			
Sometime	102	75	177			

“χ² “ = Chi-square

“*” = Significant at *p* < 0.05

### Lifestyle, risk factors and occurrence of oral pathogens

Lifestyle habits such as consumption of sugary snacks/drinks, smoking, alcohol intake, sharing toothbrushes, and wearing dental appliances were not significantly associated with presence of oral pathogens (Table 4).

**Table 4:** Association between lifestyle factors and occurrence of oral pathogens

Variables	Negative	Positive	Total	χ²	Df	*p* value
Sugary snack/drink consumption						
Daily	56	32	88	0.26	2	0.89
Occasionally	35	24	59			
Rarely	96	57	153			
Smoking/tobacco use						
Yes	19	15	34	0.52	1	0.47
No	168	98	266			
Alcohol consumption						
Yes	24	20	44	1.16	1	0.28
No	163	93	256			
Sharing toothbrush						
Yes	22	21	43	2.40	1	0.12
No	165	92	257			
Wearing dental appliances						
Yes	22	20	42	5.79	2	0.06
No	167	91	258			

“χ² “ = Chi-square

“*” = Significant at *p* < 0.05

### Oral health history and occurrence of oral pathogens

Table 5 indicates that persistent bad breath (χ² = 18.26, *p* < 0.01) and presence of mouth sores/ulcers (χ² = 7.51, *p* = 0.01) were significantly associated with occurrence of oral pathogens. Other conditions, including gum bleeding, showed no statistically significant associations.

**Table 5:** Association between oral health history and occurrence of oral pathogens

Variables	Negative	Positive	Total	χ²	df	*p* value
Diagnosis history						
Gingivitis	60	40	100	8.67	4	0.07
Periodontitis	30	25	55			
Oral candidiasis	10	8	8			
Dental caries	49	30	79			
None	48	10	58			
Gum bleeding during brushing						
Yes	76	58	134	3.69	1	0.06
No	111	55	166			
Persistent bad breath						
Yes	18	32	50	18.26	1	0.00*
No	169	81	250			
Visible tooth decay/cavities						
Yes	48	38	86	2.13	1	0.15
No	139	75	214			
Mouth sores/ulcers						
Yes	29	32	61	7.51	1	0.01*
No	158	81	239			

“χ² “ = Chi-square

“*” = Significant at *p* < 0.05

### Access to care, health status and occurrence of oral pathogens

As presented in Table 6, recent antibiotic use was strongly associated with low prevalence of oral pathogens (χ² = 10.93, *p* < 0.01). Pathogens were also more prevalent among individuals with a history of chronic illness such as diabetes mellitus, HIV infection, or other long-term medical conditions (χ² = 4.10, *p* = 0.03). Frequency of dental visits and use of mouthwash showed no significant associations.

**Table 6:** Association between access to care and occurrence of oral pathogens

Variables	Negative	Positive	Total	χ²	df	*p* value
Frequency of dental visits						
Every 6 months	29	18	47	0.71	3	0.87
Once a year	39	25	64			
Only when in pain/problem	69	45	114			
Never	50	25	75			
Chronic illness (diabetes/HIV)						
Yes	2	6	8	4.10	1	0.03*
No	185	107	292			
Recent antibiotic use						
Yes	51	52	103	10.93	1	0.00*
No	136	61	197			
Use of mouthwash						
Daily	30	15	45	0.38	2	0.83
Occasionally	78	50	128			
Never	79	48	127			

“χ² “ = Chi-square

“*” = Significant at *p* < 0.05

### Predictors of oral pathogens

Logistic regression analysis identified several factors associated with the occurrence of oral pathogens (Table 7). Children below 10 years had higher odds of the pathogens detection (OR = 3.54; 95% CI: 1.21–10.31 *p* = 0.02) compared to other age groups. Female participants had significantly higher odds of pathogens detection compared to males (OR = 5.62; 95% CI: 3.35–9.41; *p* < 0.01). Similarly, individuals residing in urban areas had increased odds compared to rural residents (OR = 2.10; 95% CI: 1.26–3.50; *p* = 0.01).

**Table 7:** Predictors of oral pathogens (logistic regression model)

Variables	Negative	Positive	Total	*p* value	Odds ratio	95% CI	
						Lower	Upper
Age							
Below 10 years	14	5	19	0.02*	3.54	1.21	10.31
11–19 years	62	52	114	0.90	0.91	0.21	4.01
20–24 years	12	4	16	0.36	1.64	0.57	4.68
25–64 years	99	52	151		1		
Gender							
Female	64	66	130	0.00*	5.62	3.35	9.41
Male	123	47	170		1		
Residence							
Rural	53	43	96	0.01*	2.10	1.26	3.50
Urban	134	70	204		1		
Recent antibiotic use							
Yes	51	52	103	0.00*	2.27	1.40	3.60
No	136	61	197		1		
Chronic illness (any)							
Yes	2	6	8	0.03*	5.19	1.01	26.6
No	185	107	292		1		
Flossing practice							
Yes, daily	41	40	81	0.01*	0.52	0.30	0.90
Occasionally	53	34	87	0.18	0.66	0.36	1.22
No	93	39	132		1		
Persistent bad breath							
Yes	18	32	50	0.00*	3.71	1.95	7.05
No	169	81	250		1		
Mouth sores/ulcers							
Yes	28	32	60	0.01*	0.46	0.26	0.81
No	159	81	240		1		

“Reference category” = 1

“*” = Significant at *p* < 0.05

Recent antibiotic use was associated with higher odds of oral pathogen detection (OR = 2.27; 95% CI: 1.40–3.60; *p* < 0.01). Participants with chronic illness also had significantly increased odds (OR = 5.19; 95% CI: 1.01–26.6; *p* = 0.03), although the estimate should be interpreted with caution due to the small sample size.

Oral hygiene practices showed significant associations, with daily flossing demonstrating a protective effect (OR = 0.52; 95% CI: 0.30–0.90; *p* = 0.01). Persistent bad breath was strongly associated with increased odds of pathogens’ colonization (OR = 3.71; 95% CI: 1.95–7.05; *p* < 0.01).

Mouth sores/ulcers were associated with reduced odds (OR = 0.46; 95% CI: 0.26–0.81; *p* = 0.01), although this finding may reflect complex clinical interactions and should be interpreted cautiously.

## Discussion

### Demographic determinants

The study identified significant associations between oral pathogen prevalence and demographic factors, including age, gender, and place of residence. Higher prevalence among adolescents and adults suggests cumulative exposure to risk factors such as poor dietary habits and suboptimal oral hygiene practices over time [6].

Females exhibited a significantly higher prevalence compared to males. This may reflect differences in health-seeking behavior, hormonal influences, or oral hygiene practices, as previously reported among Nigerian women [10]. Similarly, the higher prevalence observed among urban residents may be associated with increased consumption of refined and sugary foods, as well as lifestyle-related factors linked to urbanization.

These findings indicate that demographic characteristics are important determinants of oral pathogen distribution and should be considered when designing targeted interventions.

The current study was conducted between March and August 2025, a period that spans both the late dry season and the rainy season in North-East Nigeria. Seasonal variations may influence oral health through changes in dietary patterns, water availability, and hygiene practices. For instance, increased consumption of sugary beverages during hot periods or reduced access to clean water in certain settings may affect oral hygiene behaviors and microbial colonization. Although the present study did not specifically assess seasonal variation, these factors may have influenced the observed prevalence and distribution of oral pathogens. Future studies incorporating longitudinal or seasonal analyses are recommended to better understand these effects.

### Prevalence and pathogen profile

The detection of microorganisms in oral samples does not necessarily indicate active infection but may represent colonization or part of the normal oral microbiota. Therefore, findings in this study should be interpreted as the presence of potentially pathogenic organisms rather than confirmed clinical infection.

This study found an overall prevalence of oral pathogens of 37.67% among patients presenting with oral health complaints in North-East Nigeria, indicating a considerable burden of microbial colonization. This prevalence is consistent with national estimates in Nigeria (4–40%), and reflects the broader global burden of oral diseases, which affect approximately 3.5 billion people worldwide [5, 14, 15].

The relatively high prevalence of *Staphylococcus aureus* observed in this study should be interpreted with caution. Although *S. aureus* was the most frequently isolated organism, it is not traditionally considered a primary etiological agent of common oral diseases such as dental caries or periodontitis. Instead, it is often regarded as a transient colonizer of the oral cavity.

The presence of *S. aureus* in oral samples may therefore reflect colonization rather than active infection, and could be influenced by several factors. These include possible contamination from the skin or nasal cavity, given that *S. aureus* is a common commensal organism in these sites, as well as secondary colonization of already compromised oral tissues. Additionally, prior antibiotic exposure may alter the oral microbiota and create ecological niches that favor colonization by opportunistic organisms such as *S. aureus*.

Similar findings have been reported in recent studies, suggesting that the oral cavity can serve as a reservoir for *S. aureus*, including potentially resistant strains [5, 16]. However, its exact role in oral disease pathogenesis remains unclear. Therefore, the detection of *S. aureus* in this study should be interpreted as indicative of its presence within the oral microbial community rather than definitive evidence of a primary pathogenic role.

From a microbiological perspective, the findings of this study highlight the presence of both commensal and opportunistic organisms within the oral cavity, reflecting the complex ecology of the oral microbiome. The predominance of organisms such as *Staphylococcus aureus* and *Streptococcus mutans* suggests a shift in microbial balance that may be influenced by host, behavioral, and environmental factors. This supports the concept of oral dysbiosis as a key factor in the development of oral health conditions.

Molecular characterization and virulence profiling were not performed, which limits the ability to distinguish between colonizing and pathogenic strains.

Although *Staphylococcus aureus* was the most frequently isolated organism in this study, antimicrobial susceptibility testing was not performed. As a result, the resistance profiles of the isolates could not be determined, which limits the clinical interpretation of these findings. This is particularly important given the increasing global concern regarding antimicrobial-resistant *S. aureus*, including methicillin-resistant strains (MRSA). The absence of susceptibility data restricts the ability to guide empirical treatment or assess the public health significance of these isolates. Future studies incorporating antimicrobial susceptibility testing are therefore recommended to better characterize the clinical and epidemiological relevance of oral pathogens in this setting.

The absence of classical periodontal pathogens such as *Porphyromonas gingivalis* may be attributed to the use of conventional culture-based methods, which have limited sensitivity for fastidious anaerobic organisms. These organisms often require specialized anaerobic culture conditions or molecular techniques for accurate detection.

Similarly, the absence of *Lactobacillus* species may reflect methodological limitations, including culture conditions and media selectivity, as well as the possibility that these organisms were present at levels below detectable thresholds.

### Oral hygiene practices

Oral hygiene behaviors, particularly brushing frequency and flossing, were significantly associated with the occurrence of oral pathogens. Participants who reported more frequent brushing and regular flossing had lower prevalence of pathogens, suggesting that effective mechanical plaque control reduces microbial colonization.

This finding is supported by earlier studies, and has important practical implications, as it supports the role of routine oral hygiene practices in disrupting biofilm formation and limiting the growth of pathogenic microorganisms [9, 14, 17]. However, these results should be interpreted with caution due to reliance on self-reported data, which may be affected by recall bias and social desirability bias.

### Clinical and behavioral factors

The multivariable analysis provides important insight into the determinants of oral pathogen prevalence in this study population. Female gender and young age emerged as a strong predictors of oral pathogens colonization, which is consistent with previous studies suggesting gender and age-related differences in oral health behavior, hormonal influences, and healthcare utilization [10].

The higher odds observed among urban residents contrast with some earlier findings and may reflect lack of access to oral healthcare services, lower awareness of oral hygiene practices, and socioeconomic factors.

Interestingly, recent antibiotic use was associated with increased odds of oral pathogen detection. This finding may reflect inappropriate or incomplete antibiotic use, which can disrupt the normal oral microbiota and promote colonization by opportunistic or resistant organisms. This highlights the importance of antimicrobial stewardship in the study setting.

Chronic illnesses such as HIV, diabetes etc., were also associated with increased risk, although the small number of affected participants limits the precision of this estimate. Individuals with chronic conditions may have compromised immunity or altered oral environments that predispose them to microbial colonization.

Oral hygiene practices played a significant protective role, with daily flossing associated with reduced odds of infection. This supports the role of effective plaque control in preventing microbial accumulation and maintaining oral health.

Persistent bad breath showed a strong association with oral pathogen presence, suggesting that it may serve as a clinical indicator of underlying microbial imbalance. In contrast, the observed reduced odds associated with mouth sores/ulcers may reflect reverse causation or differences in clinical presentation and warrants further investigation. These findings are supported by several previous studies in other regions of the country [7, 8, 10].

The interpretation of some regression estimates, particularly for chronic illness, should be approached with caution due to the small sample size and wide confidence intervals.

### Public health and clinical implications

The findings of this study have important implications for oral health policy and practice in North-East Nigeria. The high prevalence of oral pathogens underscores the need for strengthened oral health education programs, particularly those promoting regular tooth brushing and flossing.

Targeted interventions focusing on high-risk groups, including adolescents, females, and urban populations, may be especially beneficial. In addition, improving access to preventive dental services and integrating oral health into primary healthcare systems could help reduce the burden of oral pathogens in the region.

Furthermore, the observed relationship between antibiotic use and pathogen prevalence highlights the importance of antimicrobial stewardship programs to prevent misuse and limit the emergence of resistant organisms.

### Limitations and strengths of the study

This study has several limitations that should be considered when interpreting the findings. First, the study employed a facility-based sampling approach, recruiting participants presenting with oral health complaints. This may introduce selection bias and limit the generalizability of the findings to the wider community.

Second, the cross-sectional design precludes causal inference, and the observed associations should be interpreted as relationships rather than cause–effect links.

Third, the study relied on conventional culture-based microbiological methods, which may not detect fastidious or anaerobic organisms, thereby underestimating the diversity of the oral microbiota.

Fourth, the detection of microorganisms in oral samples does not necessarily indicate active infection, as some organisms may represent normal flora or transient colonizers.

Additionally, antimicrobial susceptibility testing was not performed, limiting the clinical applicability of the findings, particularly for organisms such as *Staphylococcus aureus*.

Some subgroup analyses, such as chronic illness, involved small sample sizes, which may reduce the precision and stability of the estimated associations.

Finally, the study did not account for seasonal variations in oral health behaviors or environmental factors, which may influence the prevalence and distribution of oral microorganisms.

Despite these limitations, this study has several notable strengths. It provides important baseline data on the prevalence and distribution of oral microorganisms among patients in North-East Nigeria, a region with limited existing data in this area.

The study combined microbiological analysis with detailed sociodemographic and behavioral data, allowing for the assessment of multiple determinants of oral microbial presence.

The relatively large sample size enhances the reliability of the findings and enables meaningful subgroup analysis.

Furthermore, the use of standardized sample collection and laboratory procedures improves the consistency and reproducibility of the results.

Overall, the study contributes valuable insight into the epidemiology of oral microorganisms and highlights key factors associated with their presence in a resource-limited setting.

## Conclusion

This study highlights a substantial burden of oral pathogens among patients presenting with oral health complaints in North-East Nigeria, with over one-third of participants harboring at least one pathogenic organism. *Staphylococcus aureus* and *Streptococcus mutans* were the predominant isolates, underscoring the dual importance of both opportunistic and cariogenic pathogens in oral colonization. Significant associations with gender, residence, oral hygiene practices, and indicators such as persistent bad breath and mouth ulcers emphasize the multifactorial nature of oral health risks in the region. Importantly, frequent brushing and regular flossing are associated with lower prevalence of oral pathogens, supporting the need for intensified oral health promotion campaigns. The findings call for community-based interventions, improved access to dental care, and integration of oral health into broader public health strategies.

## Recommendations

Based on the findings of this study, the following recommendations are proposed:

1. There is a need to strengthen oral health education programs in the study area, with emphasis on proper oral hygiene practices such as regular tooth brushing and flossing.
2. Targeted interventions should focus on high-risk groups identified in this study, including females and individuals residing in urban areas.
3. Community-based oral health promotion and improved access to preventive dental services are recommended to reduce the burden of oral microbial colonization.
4. Healthcare providers should emphasize routine oral hygiene counseling during clinical encounters and encourage early presentation for oral health concerns.
5. Future studies should adopt community-based sampling strategies to provide more generalizable estimates of oral microbial prevalence.
6. There is a need for studies incorporating molecular diagnostic techniques to better characterize the oral microbiome, including fastidious and anaerobic organisms.
7. Additionally, antimicrobial susceptibility testing should be included to assess resistance patterns and enhance the clinical relevance of findings.
8. Longitudinal studies are also recommended to explore temporal and seasonal variations in oral microbial dynamics.

## Supplementary Information

Supplementary Material 1.

## Data Availability

All information are contained in the manuscript. Additional data are obtainable from the corresponding author, on reasonable request.
