# Supplementary material for: Determinants of oral pathogen prevalence in North-East Nigeria: a cross-sectional study
Source: BMC Oral Health. 2026 Jun 1;26:1587. doi: 10.1186/s12903-026-08774-w (PMC13504816; doi:10.1186/s12903-026-08774-w)
Supplement: Supplementary file 1 [file 12903_2026_8774_MOESM1_ESM.docx]

**FEDERAL UNIVERSITY OF HEALTH SCIENCES, AZARE, BAUCHI STATE, NIGERIA**

**Department of Microbiology**

**QUESTIONNAIRE**

Dear Respondent, We are conducting a study titled “**DETERMINANTS OF ORAL PATHOGEN PREVALENCE IN NORTH-EAST NIGERIA: A CROSS-SECTIONAL STUDY”**. Participation is voluntary, and all information provided will be kept confidential and used solely for research purposes. Your honest answers are highly valuable in making this study successful. Thank you for your time and cooperation.

***Please tick on the appropriate option***

## SECTION A: SOCIO-DEMOGRAPHIC INFORMATION

1. Age group: Below 10 years [ ] 11–19 years [ ] 20–24 years [ ] 25–64 years [ ]

2. Sex: Male	[ ]	Female	[ ]

3. Place of residence: Rural	[ ] Urban	[ ]

## SECTION B: ORAL HYGIENE PRACTICES

4. Mouth cleaning material used: Toothbrush and toothpaste	[ ] Chewing stick [ ]
 Charcoal/ash	[ ]

5. Frequency of tooth brushing: Once daily	[ ]	Twice daily	[ ] More than twice daily	[ ]

6. Toothbrush replacement frequency: Monthly	[ ]	Every 3 months	[ ]	Every 6 months	[ ]	Only when worn out	[ ]

7. Flossing practice: No	[ ]	Occasionally	[ ]	Yes, daily	[ ]

8. Mouth rinsing after meals: Always	[ ]	Sometimes	[ ]	Never	[ ]

## SECTION C: LIFESTYLE AND RISK FACTORS

9. Consumption of sugary snacks/drinks: Daily	[ ]	Occasionally	[ ]	Rarely	[ ]

10. Smoking / tobacco use: Yes	[ ]	No	[ ]

11. Alcohol consumption: Yes	[ ]	No	[ ]

12. Sharing toothbrush: Yes	[ ] No	[ ]

13. Wearing dental appliances: Yes	[ ] No	[ ]

## SECTION D: ORAL HEALTH HISTORY

14. Previous diagnosis: Gingivitis	[ ] Periodontitis	[ ] Oral candidiasis	[ ] Dental caries	[ ] None	[ ]

15. Gum bleeding during brushing: Yes	[ ] No	[ ]

16. Persistent bad breath: Yes	[ ] No	[ ]

17. Visible tooth decay/cavities: Yes	[ ] No	[ ]

18. Mouth sores / ulcers: Yes	[ ] No	[ ]

## SECTION E: ACCESS TO CARE AND HEALTH STATUS

19. Frequency of dental visits: Every 6 months	[ ] Once a year	[ ] Only when in pain/problem	[ ] Never	[ ]

20. History of chronic illness: Yes	[ ] No	[ ]

21. Recent antibiotic use: Yes	[ ] No	[ ]

22. Use of mouthwash: Daily	[ ] Occasionally	[ ] Never	[ ]
